# Complication avoidance protocols in endoscopic pituitary adenoma surgery: a retrospective cohort study in 514 patients

**DOI:** 10.1007/s11102-021-01167-y

**Published:** 2021-07-02

**Authors:** Jai Deep Thakur, Alex Corlin, Regin Jay Mallari, Samantha Yawitz, Amalia Eisenberg, Walavan Sivakumar, Chester Griffiths, Ricardo L. Carrau, Sarah Rettinger, Pejman Cohan, Howard Krauss, Katherine A. Araque, Garni Barkhoudarian, Daniel F. Kelly

**Affiliations:** 1 Pacific Neuroscience Institute, 2125 Arizona Ave, Santa Monica, CA 90404 USA; 2 Saint John’s Cancer Institute (Formerly John Wayne Cancer Institute), Providence Saint John’s Health Center, Santa Monica, CA USA; 3 University of South Alabama, Mobile, AL USA; 4 Ohio State Wexner Medical Center, Columbus, OH USA

**Keywords:** Complication, Pituitary adenoma, Endoscopic endonasal, Acromegaly, Cushing’s disease, Prolactinoma, Hypopituitarism, Length of stay, Hospital readmission

## Abstract

**Purpose:**

To evaluate the impact of using consistent complication-avoidance protocols in patients undergoing endoscopic pituitary adenoma surgery including techniques for avoiding anosmia, epistaxis, carotid artery injury, hypopituitarism, cerebrospinal fluid leaks and meningitis.

**Methods:**

All patients undergoing endoscopic adenoma resection from 2010 to 2020 were included. Primary outcomes included 90-day complication rates, gland function outcomes, reoperations, readmissions and length of stay. Secondary outcomes were extent of resection, short-term endocrine remission, vision recovery.

**Results:**

Of 514 patients, (mean age 51 ± 16 years; 78% macroadenomas, 19% prior surgery) major complications occurred in 18(3.5%) patients, most commonly CSF leak (9, 1.7%) and meningitis (4, 0.8%). In 14 of 18 patients, complications were deemed preventable. Four (0.8%) had complications with permanent sequelae (3 before 2016): one unexplained mortality, one stroke, one oculomotor nerve palsy, one oculoparesis. There were no internal carotid artery injuries, permanent visual worsening or permanent anosmia. New hypopituitarism occurred in 23/485(4.7%). Partial or complete hypopituitarism resolution occurred in 102/193(52.8%) patients. Median LOS was 2 days; 98.3% of patients were discharged home. Comparing 18 patients with major complications versus 496 without, median LOS was 7 versus 2 days, respectively p < 0.001. Readmissions occurred in 6%(31/535), mostly for hyponatremia (18/31). Gross total resection was achieved in 214/312(69%) endocrine-inactive adenomas; biochemical remission was achieved in 148/209(71%) endocrine-active adenomas. Visual field or acuity defects improved in 126/138(91.3%) patients.

**Conclusion:**

This study suggests that conformance to established protocols for endoscopic pituitary surgery may minimize complications, re-admissions and LOS while enhancing the likelihood of preserving gland function, although there remains opportunity for further improvements.

## Introduction

**Fig. 1:**
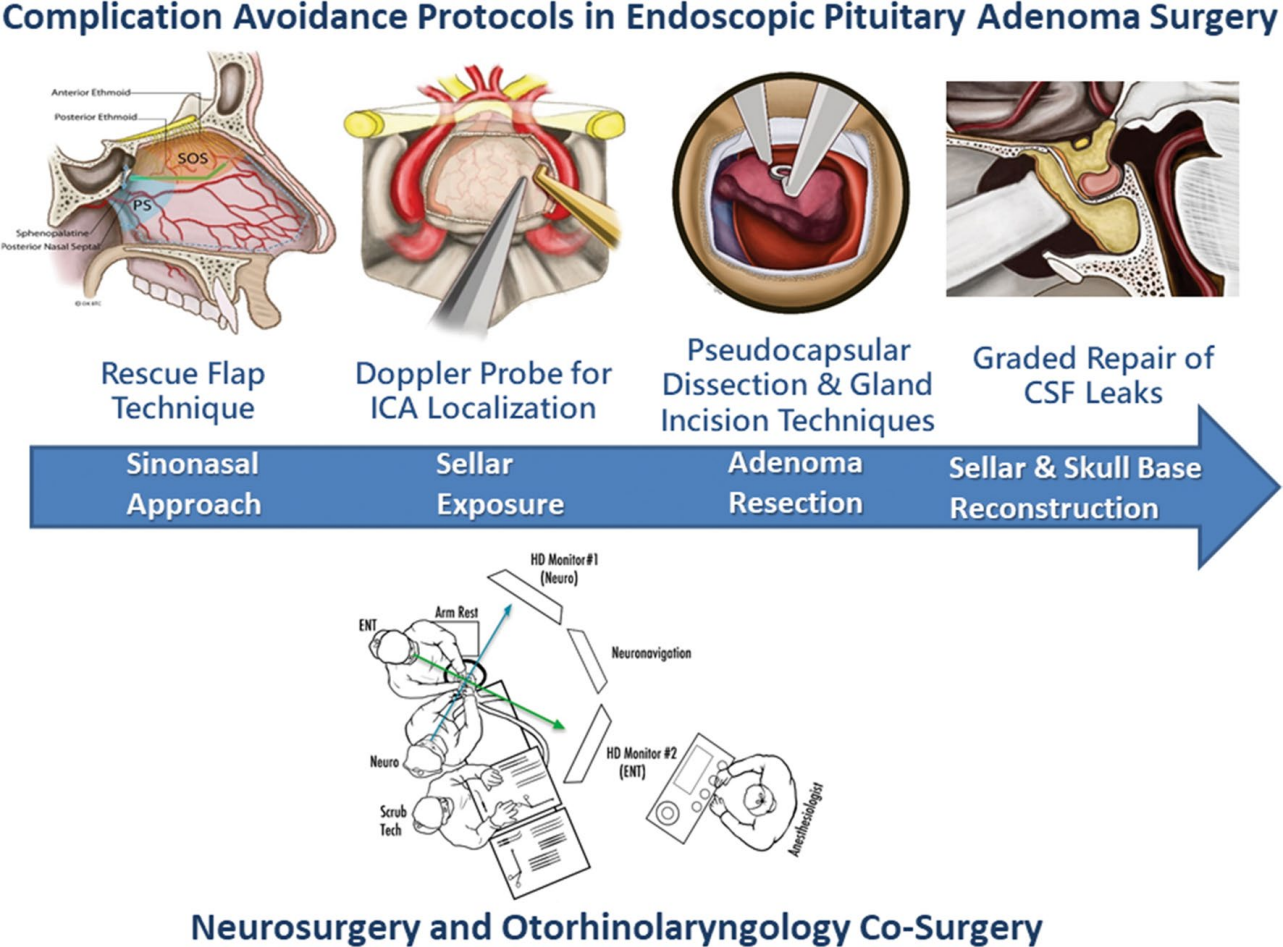
Complication avoidance protocols and operative suite setup for endoscopic pituitary adenoma surgery with neurosurgery and otorhinolaryngology (ENT). Critical surgical phases include (1) the sinonasal approach with creation of rescue flaps, which aim to avoid anosmia and delayed post-operative epistaxis, (2) sellar exposure and Doppler localization of the carotid arteries to avoid ICA injury, (3) tumor removal with pseudocapsular dissection and gland incision techniques to maximize resection/remission rates and avoid new gland injury, and (4) sellar and skull base reconstruction with graded repair protocol to avoid CSF leaks and meningitis

Pituitary adenomas are the third most common primary intracranial tumor accounting for approximately 15% of all intracranial tumors, and surgical resection is considered first-line therapy for all adenoma subtypes except prolactinomas [1, 2]. Transsphenoidal pituitary surgery dates back over a century, becoming well-established with overall excellent outcomes in the 1970s [3]. Numerous advances since the 1990s appear to have improved the safety and efficacy of this procedure [4–16]. Arguably, the most important advance is the application of endoscopy which has become the predominant visualization tool and replaced the operating microscope at many centers [4, 5, 17]. This transition has fueled collaboration between otolaryngologists and neurosurgeons in endoscopic skull base surgery, resulting in expanded knowledge of skull base and parasellar anatomy and refined surgical approaches, resection and reconstruction techniques (Fig. 1). Since 2013, most pituitary adenoma operations in the United States are performed endoscopically, and the endonasal route is increasingly the preferred route for many midline skull base tumors such as craniopharyngiomas, meningiomas and clival chordomas [18]. Other important technological and technique innovations include (1) the “rescue flap” technique which spares the sphenopalatine vascular pedicle along the nasal septum as well as the septal olfactory strip to avoid postoperative epistaxis and anosmia [15, 16]; (2) Doppler probe localization of the parasellar carotid arteries for avoiding ICA injury [8], (3) the pseudocapsular dissection technique for maximizing chances of remission/resection as well as gland sparing and incising techniques for preserving pituitary gland function [7, 19], and (4) graded repair technique for improved skull base closure, including the selective use of pedicle nasoseptal flaps to avoid CSF leaks and bacterial meningitis [9–11]. From an organizational aspect, the concept of multidisciplinary *pituitary centers of excellence* has been promoted to better integrate collaboration among neurosurgeons, endocrinologists, otolaryngologists, neuro-ophthalmologists and radiation oncologists, to optimize overall management [12, 13]. Consensus guidelines have also been produced by endocrinology and neurosurgical experts in the field that have provided recommendations on optimal multidisciplinary management of specific adenoma subtypes such as acromegaly and Cushing’s disease [20, 21].

It is our hypothesis that endoscopic pituitary adenoma surgery when consistently performed using these four surgical techniques of (1) rescue flap approach technique, (2) Doppler probe carotid artery localization, (3) pseudocapsular resection and gland sparing tumor resection techniques, and (4) graded repair of CSF leaks, is associated with low complication rates, readmissions, reoperations and short LOS, with high rates of gland preservation or recovery, and adenoma resection/remission rates (Fig. 1). We also hypothesize that over time, increasing experience leads to further reductions in complications and LOS. A 10-year retrospective review of incorporating these complication avoidance techniques was undertaken with a focus on preventable complications and those leading to permanent neurological sequelae.

## Methods

### Study design and patient cohort

This study was approved by the Saint John’s Cancer Institute IRB (IRB#JWCI-19-1101). From April 2010 through August 2020, all patients undergoing endoscopic adenoma removal at Saint John’s Health Center with minimum 3-month follow-up were identified. A prospectively maintained database was retrospectively analyzed. As recently described, data collection included surgical indications, operative notes, hormonal and visual data, MRI, histopathology, and complications [22]. All procedures were performed by one of two neurosurgeons with one of three otolaryngology co-surgeons in non-overlapping fashion.

### Preoperative assessment

All patients underwent complete pituitary hormonal testing, with additional provocative testing as needed for patients with endocrine-active adenomas, as well as pituitary MRI without and with gadolinium. Visual fields and visual acuity were tested in all patients and those with macroadenomas causing chiasmal or optic nerve compression typically underwent formal visual field and acuity testing. Olfactory function was assessed preoperatively and re-assessed at least 6 weeks post-surgery using Brief Smell Identification Test (BSIT, Sensonics Corporation; Haddon Heights, NJ, USA).

### Pituitary hormonal testing

As previously described, pituitary gland function was evaluated with pre- and postoperative tests (at least 6 weeks post-surgery) along corticotroph, thyrotroph, gonadotroph and somatotroph axes, resolution of stalk-compression hyperprolactinemia in non-prolactinomas, and new transient (less than 6 weeks) or permanent diabetes insipidus (DI) [19, 22–24]. Since many patients came with testing from outside laboratories, results were interpreted based on provided reference ranges. Stalk-compression hyperprolactinemia (serum prolactin > 20 ng/ml for men and > 25 ng/ml for women) and its recovery were reported as separate outcomes. Although IGF-1 and growth hormone (GH) were collected on all patients, somatotroph axis analysis was excluded from the study given infrequent use of stimulation testing to assess for pre- and post-operative GH deficiency. Improvement in any of the three anterior gland axes was based on normalization of values or being weaned off replacement therapy, at 6 weeks or greater post-surgery. Patients were diagnosed as having DI if urine specific gravity was 1.005 or less and urine volume was greater than 200 mL/h for at least 3 consecutive hours with a serum Na > 145 mEq/l.

### Surgical technique and protocol

After a preoperative surgical time-out and induction of total intravenous anesthesia [25], the patient is typically positioned on a horse-shoe head holder [15, 19, 22, 26]. The abdomen is prepped for possible fat graft. Neuro-navigation (Stryker Inc, Kalamazoo, MI; or BrainLab, Munich, Germany) and micro-Doppler probe (Koven, St. Louis, MO; or Vascular Technologies Inc, Beverly, MA) are used in all cases [8]. Somatosensory evoked potential monitoring with direct cranial nerve stimulation is used in select cases when CS exploration is anticipated. Foley urinary catheter placement are only used in select patients with anticipated case duration over 4 h. Surgery is performed using a binostril, two-surgeon approach with a neurosurgeon and otolaryngologist initially with a 0° 4-mm endoscope. (Karl Storz-America, El Segundo, CA) [15, 19, 22, 26]. Key details of the overall protocol are as follows.

### Sinonasal approach, sphenoidotomy and sellar exposure

A bilateral rescue flap technique is performed for the great majority of adenoma cases with the goal of sparing the sphenopalatine arteries and the septal olfactory strips to minimize chances of permanent anosmia; pedicled nasoseptal flaps are rarely used [15, 26]. Middle turbinates are out-fractured, not removed [15]. A wide sphenoidotomy is performed. The bony sellar removal is tailored to tumor pathology. The adequacy and safety of the sellar opening are guided by navigation and Doppler ultrasound of the cavernous segment of the internal carotid arteries [8].

### Adenoma resection and sellar closure

A selective adenomectomy with a pseudocapsular technique is attempted to improve chances of GTR and preserve pituitary gland function. Selective gland incisions and/or resections are performed as needed to optimize tumor access and preserve healthy gland [19]. Angled endoscopes (30° and 45°) are often used for tumor resection in or along the CS, and at superior tumor surface-gland interface. A multilayer sellar closure and graded CSF leak repair is performed as recently described [26].

### Post-op surveillance and activity

Patients are typically admitted to a step-down unit (non-ICU bed). On POD#1, a pituitary MRI is obtained, and hormonal function and electrolytes are assessed for adrenal insufficiency, DI and early remission for endocrine-active adenomas on POD 1–2 [17, 22, 26, 27]. Early ambulation is encouraged, and most patients are discharged by POD 2. All patients have a provocative “tilt test” prior to discharge to assess for an unrecognized CSF leak [10, 22, 26].

Discharge instructions include (1) a 1-L fluid restriction for 7 days post-surgery to help prevent delayed hyponatremia [28] as well as liberal use of table salt in meals and electrolyte-rich drinks (e.g. Gatorade) unless in DI at time of discharge, (2) three daily nasal saline irrigations beginning on post-operative day 3 if no nasal packing or on post-operative day 6 (after packing is removed), (3) increasing ambulation during first post-operative week but no blowing the nose, bending over, or heavy lifting; driving is allowed after 12–14 days and gradual return to full activities including exercise by 3 weeks post-surgery.

Follow-up includes otolaryngology and neurosurgery clinic visits within 10 days of surgery and serum sodium on POD 5–7 to monitor for delayed hyponatremia and endocrinology follow-up is within 6 weeks of surgery [28, 29]. Subsequent MRI and hormonal testing are diagnosis-dependent but at a minimum occur at 3 months post-surgery. Long-term follow-up for at least 10 years is attempted in all patients, at least annually for the first 5 years and then every 18 months to 2 years thereafter, but is dependent upon resection or remission status of each patient [22].

### Primary outcome variables

All 90-day complications were noted including (1) *sinonasal approach, sphenoidotomy and sellar exposure-related complications* of anosmia, epistaxis and carotid artery or other vascular injury; (2) *tumor resection related complications* of pituitary gland injury (new hypopituitarism), visual acuity or visual field deterioration, new diplopia, postoperative hematoma; (3) *sellar and skull base closure related complications* of CSF leak and meningitis; (4) *systemic non-neurological complications* of pulmonary embolism (PE), deep vein thrombosis (DVT), myocardial infarction (MI); (5) *minor complications* of delayed hyponatremia and abdominal fat graft site hematoma/infection. All repeat operations and 30-day readmissions were documented as were hospital length of stay (LOS) and discharge disposition. Preventable complications are defined as those which could have been avoided.

### Secondary outcome variables

For endocrine-active adenomas, early remission was defined as previously cited [17, 22, 23, 30]: (i) acromegaly, postoperative day (POD) 1–2 GH level ≤ 1 ng/ml and a subsequent normal IGF-I at least 3 months post-surgery; (ii) Cushing’s disease (CD), POD 1–2 morning cortisol level < 5mcg/dl and needing cortisol replacement for at least 3 months post-surgery; (iii) prolactinoma, POD 1–2 serum PRL level < 10 ng/dl and normal prolactin(< 20 ng/ml for men and < 25 ng/ml for women) at least 3 months post-surgery without dopamine-agonist therapy; (iv) thyrotropinoma: normal TSH and free T4 at least 3 months post-surgery. Patients undergoing repeat surgery within 3 months were considered as one entity for remission status.

For endocrine-inactive adenomas, resection was categorized as (i) gross total resection (GTR, no tumor seen intraoperatively and in early and 3-month postoperative MRI), (ii) near total resection (NTR):90–99% resection based on postoperative MRI, (iii) subtotal resection (STR): ≤ 89% resection) [17, 22]. Post-operative visual acuity and field outcomes were categorized as resolved, improved, persistent or worsened at 3 months or greater post-surgery. Preoperative and postoperative status of patients with headaches were categorized as: resolved, improved, persistent or worsened [22]. As recently described, CS invasion was deemed present if preoperative coronal MRI showed Knosp Grade 4 invasion and/or if the intraoperative endoscopic view showed cavernous sinus invasion as documented in surgeons’ operative notes [6, 22, 31].

### Statistical analysis

Demographics, patient and tumor factors and other categorical variables were compared by bivariate analysis (Chi-square or Fisher exact test). Intergroup differences between nonparametric medians were compared using Kruskal–Wallis testing, with two-sided p-values with p < 0.05 considered statistically significant. Statistical analysis was performed with IBM SPSS Statistics, version 26, Chicago.

## Results

### Patient demographics and clinical presentation

Over the study period of April 2010–August 2020, there were a total of 820 endoscopic endonasal operations for tumor or cyst. As shown in Table 1, of these 820 operations, 549 (67%) operations were performed in 514 patients for pituitary adenoma (52.3% females, mean age 51 ± 16 years; 78.0% (428) macroadenomas). In total, 100 (19%) had prior surgery (82% by another surgical team). The most common clinical indications for surgery were for hypopituitarism, endocrine-active adenomas and visual field and/or acuity deficits. Mean follow-up for the entire cohort was 27.4 ± 26.6 months.

**Table 1:** Clinical features and operative details of 514 patients with pituitary adenomas undergoing 549 operations

	N (%)
Mean age	51 ± 16 years
Female	269 (52.3%)
Male	245 (47.7%)
Endocrine-inactive adenoma patients	305 (59.3%)
Endocrine-inactive operations	312 (56.8%)
Endocrine-active adenoma patients	209 (40.7%)
Endocrine-active operations	237 (43.2%)
Cushing’s disease	102 (18.6%)
Acromegaly	60 (10.9%)
Prolactinoma	69 (12.6%)
TSH-secreting	3 (0.5%)
Mixed GH-prolactin or GH-TSH secreting	3 (0.5%)
*Clinical presentation and Surgical Indications in 514 patients*	
Hypopituitarism	215
Endocrine-active adenoma	209
Vision field or acuity deficit	145
Headaches^a^	132
Incidental^a^	89
Apoplexy	59
First-time operation	414 (75.4%)
Redo operation^#^	135 (24.6%)
Standard approach to sella	337 (61.4%)
Extended approach to parasellar area	212 (38.6%)
Intraoperative CSF leak in 549 operations	
Grade 0	268 (48.8%)
Grade 1	142 (25.9%)
Grade 2	104 (18.9%)
Grade 3	35 (6.4%)
Skull base reconstruction	
Nasoseptal flap usage	12 (2.2%)
Lumbar CSF diversion	0

^a^All patients with only headaches or an incidentally discovered adenoma, had at least one other surgical indication: endocrine-active adenoma, hemorrhage/apoplexy, visual deficit, hypopituitarism, severe gland compression, tumor recurrence, large or invasive macroadenoma, tumor growth on serial MRIs, concern for metastatic carcinoma, patient preference. **#** In total, 100 patients had a prior surgery

### Primary outcomes

*Surgical and nonsurgical complications:* In total, 25 major complications occurred in 18 patients and are detailed in Table 2 by complication type and in Table 3 by individual patient. There was one unexplained death, one non-carotid vascular injury, and one sellar hematoma leading to permanent cranial neuropathy (rate 0.2%) all occurring prior to 2016. One additional neurological decline occurred in a patient with a giant invasive adenoma with prior surgery. There were no carotid artery injuries or new anosmia. One patient had transient mild visual acuity decline that resolved after the 90-day follow-up period. Epistaxis from a sphenopalatine branch artery occurred in 3 (0.6%) patients requiring treatment. Two (0.4%) patients sustained a PE but there were no DVTs or MIs. The most common minor surgical complications were transient DI, sinusitis and new hyposmia.

**Table 2:** Primary outcomes: complications, length of stay, disposition, 30-day readmissions and reoperations

	N (%)
*Major surgical complications* (N = 25 in 18 patients)^a^	
Death	1/514 (0.2%)
Carotid artery injury	0
Other major vascular injury	1/514 (0.2%)
Permanent major vision decline	0
Other new neurological deficit	4/514 (0.8%)
Postoperative sellar hematoma	1/514 (0.2%)
Post-operative CSF leak	9/514 (1.7%)
Bacterial meningitis	4/514 (0.8%)
Epistaxis requiring treatment (from sinonasal source)	3/514 (0.6%)
New anosmia	0
Pulmonary embolus	2/514 (0.04%)
Deep vein thrombosis	0
Myocardial infarction	0
*New hypopituitarism*	23/485 (4.7%)
Anterior hypopituitarism	17/485 (3.5%)
Persistent DI (5 of which also had new anterior hypopituitarism)	11/485 (2.3%)
*Minor surgical complications*	
New hyposmia	8/514(1.6%)
ENT intervention for chronic sinusitis	16/514 (3.1%)
Fat graft site hematoma	2/514 (0.4%)
Transient diabetes insipidus	62/514 (12.1%)
*Median length of stay*	2 days
Discharge to home^b^	526/535 (98.3%)
Discharge on POD#1^b^	56/535 (10.5%)
Discharge after POD#4^b^	27/535 (5.0%)
*Patients needing reoperation for residual tumor: 31 of 514 patients*	
Patients needing 1 reoperation	27
Patients needing 2 reoperations	4
Total operations	549

^a^Of 18 patients with major complications, 5 had multiple complications

^b^Total admissions were 535 in 514 patients

**Table 3:** Details of 18 patients with major complications

Year	Tumor Type	Prior surgery	Complication	Total LOS	Intervention required	Preventable	Long-term sequelae
1st Half of Cohort, N = 257 Patients							
2010, 71F	Endo-Inactive	No	CSF leak	5	Return to OR	Yes	No
2011, 43M	Endo-Inactive	No	CSF leak, meningitis	11	Lumbar CSF diversion, antibiotics	Yes	No
2011, 57M	Endo-Inactive	Yes	Hematoma, CN3 palsy	5	Return to OR	Yes	Yes
2011, 48F	Cushing’s	No	Basilar artery pseudoaneurysm, CSF leak, meningitis, coma	28	Multiple reoperations	Yes	Yes
2012, 54M	Endo-Inactive	No	CSF leak	5	Return to OR	Yes	No
2013, 44F	Cushing’s	No	Meningitis	11	Antibiotics	No	No
2013, 66M	Giant Endo-Inactive	Yes	Epistaxis, mild transient decrease in visual acuity	7	Cautery, packing, transfusion	Yes	No
2014, 63M	Acromegaly	No	Epistaxis	3	Cautery, packing	Yes	No
2014, 67F	Endo-Inactive	No	CSF leak, meningitis	17	Lumbar drain, antibiotics	Yes	No
2014, 60F	Cushing’s	No	CSF leak	8	Return to OR	Yes	No
2015, 28F	Endo-Inactive	No	Epistaxis	2	Cautery and injection	Yes	No
2nd half of cohort, N = 257 patients							
2015, 61M	Endo-Inactive	No	Progressive vasculitis, hemiparesis, death	3	None	No	Yes
2017, 54F	Cushing’s	Yes	Pulmonary embolism	11	Readmission, heparin and coumadin	Yes	No
2018, 62M	Giant Endo-Inactive	Yes	Oculoparesis, anterograde amnesia	10	None	No	Yes
2019, 46F	Cushing’s	No	CSF leak	3	Return to OR	Yes	No
2019, 51F	Cushing’s	No	CSF leak	8	Lumbar drain	Yes	No
2019, 46M	Endo-Inactive	No	CSF leak	4	Return to OR	Yes	No
2020, 34M	Cushing’s	No	Pulmonary embolism	8	Readmission, heparin and coumadin	No	No

^a^Note that 10 of 18 (56%) patients had either Cushing’s disease, a giant adenoma and/or prior surgery. The 2^nd^ half of the study started in May 2015

### CSF leaks and meningitis

An intra-operative CSF leak was observed in 51.2% cases. Nasoseptal flaps were utilized in 12 (2.2%) cases and planned lumbar spinal drains in none. A post-operative CSF leak occurred in 9 (1.7%) patients, managed by reoperation in 6 and lumbar spinal drainage in 3. Bacterial meningitis occurred in 4 (0.8%) patients, all of whom fully recovered.

### Pituitary gland function decline and improvement

Hormonal data was available for 485/514 (94.4%) patients; new hypopituitarism occurred in 23/485(4.7%): 17(3.5%) with new anterior hypopituitarism and 11(2.3%) with persistent DI. Pituitary gland function improved in at least one axis in 102/193 (52.8%) patients with preoperative hypopituitarism, including resolution of stalk compression hyperprolactinemia in 68/78 (87.2%) patients (Table 4).

**Table 4:** Secondary outcomes: adenoma resection/remission rates, vision, headache and gland function improvement

Gross total resection: endocrine-inactive adenoma (in 312 operations)	214/312 (68.6%)
Endocrine remission: endocrine-active adenoma (in 209 patients)	148/209 (70.8%)
Improvement of preoperative vision, headache and gland function	
Vision improvement: complete 34.1%; partial 57.2%	126/138 (91.3%)
Headache resolution: complete 82.6%; partial 15.2%	129/132 (97.7%)
Pituitary gland function improvement (at least one axis improved)	102/193 (52.8%)
Gonadal axis recovery	71/107 (66.4%)
Thyroid axis recovery	38/97 (39.2%)
Adrenal axis recovery	10/97 (10.3%)
Stalk compression hyperprolactinemia resolution	68/78 (87.2%)

### Preventable and nonpreventable complications

Of 18 patients who sustained major surgical complications, 14 were deemed preventable including 9 patients with CSF leaks (2 of whom had meningitis and one had a basilar artery injury), 3 with epistaxis and one of two patients with PE (Tables 4, 5, 6). The four patients with nonpreventable complications are described here. One patient with Cushing’s disease developed bacterial meningitis without a post-operative CSF leak, treated successfully with antibiotics. One patient with Cushing’s disease, multiple prior DVTs and sagittal sinus thrombosis, developed a post-operative PE. He had an inferior vena cava filter placed preoperatively and all DVT prophylaxis protocols were strictly followed but he sustained a PE diagnosed on POD#10 on a follow-up visit after discharge home. He was treated successfully with anticoagulation. One patient with a recurrent giant adenoma developed anterograde amnesia and oculoparesis after uneventful tumor debulking. One patient died of unclear causes (described below).

**Table 5:** Endocrine-inactive adenomas: bivariate factors related to gross total resection

Total: 312 operations in 305 patients	GTR rate	p value
Maximal tumor diameter		** < 0.001**
< 20 mm (111)	87/111 (78.4%)	
20–29 mm (126)	91/126 (72.2%)	
30 or more (75)	36/75 (48.0%)	
Giant adenoma (18)	3/18 (16.7%)	** < 0.001**
Non-giant adenoma (294)	211/294 (71.8%)	
Cavernous sinus invasion (121)	42/121 (36%)	** < 0.001**
No cavernous sinus invasion (191)	172/191 (90%)	
Redo operation (66)	26/66 (39.4%)	** < 0.001**
No prior operation (246)	188/246 (76.4%)	

p values of < 0.05 are considered signficant

^a^Factors of age and intraoperative CSF leak were not significant factors

**Table 6:** Endocrine-active adenomas: bivariate factors related to early remission

Total: 209 patients	Remission rate	p value
Cavernous sinus invasion (n = 73)	44/73 (60.3%)	**0.037**
No cavernous sinus invasion (n = 136)	104/136 (76.5%)	
Redo operation (n = 42)	23/42 (54.7%)	**0.005**
No prior operation (n = 167)	125/167 (74.8%)	

p values of < 0.05 are considered signficant

^a^Factors of age and tumor diameter were not significant factors

### Complications with permanent neurological sequelae

#### Death

One death occurred in a man 3 days after uneventful macroadenoma removal who presented with worsening headaches but no MRI-evidence of apoplexy. A GTR was accomplished and Grade 2 CSF leak repaired uneventfully; EBL was 150 cc. Immediate post-operative head CT showed no sellar, subarachnoid or intraparenchymal hemorrhage or pneumocephalus. He awoke normally but became obtunded over next 36 h and developed bilateral watershed infarcts on MRI. An angiogram confirmed diffuse multi-vessel cerebral vasculitis; LP showed no meningitis. An autopsy was inconclusive but suggested possible reversible vasoconstriction syndrome.

#### Vascular injury

During a repeat operation for a woman with severe Cushing’s disease and osteopenia, the basilar artery sustained an unrecognized injury with the drill that perforated a thinned clivus. CT angiography immediately post-surgery was normal, however, four days post-surgery shortly after a follow-up CT angiogram showed a delayed basilar pseudoaneurysm, the patient suffered a devastating hemorrhage leading to persistent coma.

#### Symptomatic sellar/cavernous sinus hematoma

A sellar hematoma occurred in a man with a recurrent invasive endocrine-inactive macroadenoma extending through the cavernous sinus and oculomotor triangle. Residual tumor hemorrhaged with new oculomotor nerve deficit prompting urgent reoperation; the oculomotor palsy persisted.

#### Anterograde amnesia and oculo-paresis

A man with a recurrent giant endocrine-inactive adenoma (extending into the 3rd ventricle) with surgery 9 years previously and shunted hydrocephalus, had a near-complete uneventful tumor removal. The patient awoke slowly with memory issues and oculoparesis that improved significantly over two months but ultimately required a right superior oblique tendon tuck 2 years after surgery.

### Length of stay, disposition and readmissions

Of 535 total admissions, 526 (98.3%) patients were discharged to home, 8 to rehabilitation and one died. Median LOS was 2 days; 56 (10.5%) were discharged on POD 1; 27 (5.0%) had LOS of > 4 days. Of these 27 patients with LOS > 4 days, a majority (17/27) had endocrine-active adenomas. Comparing 18 patients sustaining a major complication to 496 who did not, median LOS was 7 versus 2 days respectively, p < 0.001. Thirty-one of 535 (6%) patient admissions required hospital readmission, most commonly for delayed hyponatremia (n = 18), CSF leak (n = 4) and PE (n = 2) or headache (n = 2**)** (Table 7). Regarding hyponatremia-related readmissions, within the second half of the 10-year cohort, there have been fewer recently: 8 occurred from 2015 to 2017 and one in 2018 and none in 2019 or 2020.

**Table 7:** Reasons for readmission within 30 days of surgery

All Readmissions: 31/535 (6%)	
Hyponatremia	18
CSF leak	4
Headaches	2
Pulmonary Embolus	2
Meningitis	1
New infarct/hemorrhage	1
Suspicion of CSF leak, negative	1
Abdominal fat graft site hematoma	1
Shunt revision	1
Total	31

### Complication rates, hypopituitarism, LOS and readmissions over time

Overall, of 18 (3.5%) patients sustaining major complications, 11 (61%) were in the first half (257 patients) and 7 (39%) were in the second half (257 patients) of the study period, (p = 0.47). The CSF leak rate decreased from 6/257 (2.3%) to 3/257 (1.2%), (p = 0.5) and the meningitis rate dropped from 4/257 (1.6%) to 0 (p = 0.12) in the 1st and 2nd halves of the series, respectively. New permanent hypopituitarism occurred in 12 patients in the first half and 11 patients in second half of the series. Median LOS of 1 day increased from 4.1% in first half to 16.9% in second half of series and increased to 26% of the last 100 operations. Readmissions remained stable at 15/268 and 16/267 In first and second half of study timeline.

### Secondary outcomes

As shown in Table 4, for endocrine-inactive adenomas, in 305 patients undergoing 312 operations, GTR was obtained in 214 (68.6%), NTR in 68 (21.8%), STR in 30 (9.6%). For endocrine-active adenomas, of 209 patients early biochemical remission was achieved in 148/209 (70.8%) patients: CD (55/81) 67.9%; GH (42/56) 75.0%, prolactinoma (46/66) 69.6%; TSH (3/3) 100%; GH-prolactin (1/2) 50%; GH-TSH (1/1) 100%. Bivariate analyses of factors associated with GTR and endocrine remission are shown in Tables 5 and 6, respectively. Preoperative visual field or acuity defects improved in 126/138(91.3%), headaches resolved or improved in 129/132(97.7%).

## Discussion

### Summary of experience

In 514 patients undergoing endoscopic pituitary adenoma removal over a 10-year period, major surgical complications occurred in 18 (3.5%) patients, including 4 (0.8%) that had permanent neurological sequelae. CSF leaks were the most common preventable complication occurring in 1.7% of the cohort, followed by meningitis in 0.8%. Worsening of gland function occurred in 4.5%. Notably there were no carotid artery injuries, permanent worsening of visual acuity or visual fields and no new permanent anosmia. The complication avoidance protocol followed in this cohort was associated with a median LOS of 2 days and 98% of patients being discharged to home. Overall complication rates decreased between the first and second half of the study period.

### Complication avoidance protocols and their impact

Even prior to the advent of endoscopy over two decades ago, transsphenoidal pituitary adenoma surgery with the operating microscope was established as a relatively safe and effective procedure [3]. Adoption of the fully endoscopic approach has promoted a “*team surgery”* concept of neurosurgeons and otolaryngologists working side-by-side for pituitary and parasellar pathology although at some centers, pituitary surgery is still performed only with neurosurgeons [4, 5].

Over more than two decades, numerous groups have documented the incidence and root causes of complications in endoscopic pituitary surgery and stressed the importance of adherence to complication avoidance protocols to maximize patient safety and optimize outcomes [8, 10, 15, 23, 26, 32–38]. This collective experience by our group and others demonstrates that surgery is safe and effective for the great majority of patients, including patients undergoing reoperations, which represents 19% of our cohort. Table 8 highlights complication and resection rates in recent large endoscopic cohort studies since 2010 confirming the overall safety of this approach. However, it also reaffirms that careful attention to every stage of surgery including the sinonasal exposure, sellar opening and ICA localization, tumor resection, gland handling and sellar reconstruction is critical to achieving safe and successful outcomes.

**Table 8:** Major complication rates after endoscopic adenoma resection in recent large series

Study and Year	N and Time Period	Prior surgery %	Extent of Resection %	Mean LOS (days)	Carotid Injury %	Other Vascular injury or Stroke %	Vision Decline %	Other New CN Deficit %	CSF Leak %	Meningitis %	New Hypopituitarism Total, Anterior or Posterior Gland %	Op Hematoma %	Epistaxis %	Anosmia %	Death %
Gondim (2010)	N = 301 1998–2009	NR	GTR 71.1	NR	1.0	0	0.3	0	2.7	0.7	A 11.6, P 1.3	0.7	2.0	NR	1.0
Palluzi (2014)	N = 555 2002–2011	17.5	GTR 65.3	NR	0.3	0.2	2.5	0.6	5	0.9	A 3.1; P 2.5	1.1	1	2.1	0.2
Magro (2016)	N = 300 2002–2013	14	GTR 59	NR	0.3	NR	2.43	2.8	2.7	3.3	A 13.7; P 6.2	2	2.3	1	0.7
Eseonu (2017)	N = 275 2005–2015	16.1	Mean EOR 85.1	2.4	0.4	0.73	5.49	0.36	3.6	0.7	Total 3.3 P 0.7	0	0.36	NR	0
Kim (2018)	N = 331 2010–2016	29.3	GTR 74.9	NR	0	0	2.7	NR	2.4	5.4	Total 32.9 A 28.6 P3	2.4	NR	NR	0.3
Little (2019) (Prosp)	N = 169 2015–2017	8.3	GTR 79.3	NR	NR	NR	NR	NR	NR	NR	Total 9.7 P 2.4	NR	NR	NR	NR
Younus (2021)	N = 584	12.8	GTR 71.9	4.1	NR	0.5	3.1	1.5	0.7	0.5	NR	1.5	NR	NR	NR
Bernat (2021)	N = 269 2005–2015	13.0	GTR 46.0	NR	NR	NR	0.7	NR	2.2	NR	NR	3.0	NR	NR	0
Current Study	N = 514 2010–2020	19	GTR 69, Endocrine Remission 71%	2^a^	0	0.2	0	0.4	1.7	0.8	Total 4.7 A 3.7; P 2.1	0.2	0.6	0	0.2

All studies are retrospective except Little (2019)

*LOS* length of stay, *EOR* extent of resection, *N* number of patients, *NR* not reported, *GTR* gross total resection, *A* anterior, *P* posterior

^a^Median

Another factor in procedural safety is the consistent use of a preoperative timeout for each operation which is now considered standard of care in neurosurgery and other surgical subspecialties [39]. We have also implemented a “carotid artery injury timeout” in the last 2 years for endoscopic endonasal cases that have a higher risk of carotid artery manipulation and injury such as some adenomas with cavernous sinus invasion and previously operated and/or irradiated adenomas; fortunately we have not had any ICA injuries in this series.

Regarding sinonasal function and olfaction, the collaboration with otolaryngology has been extremely useful in the effort to maintain this important aspect of quality-of-life after endonasal surgery. The bilateral rescue flap and mucosal preservation technique strives to maintain anatomical and physiological integrity of the septal olfactory strip. The added benefit of rare middle turbinate resection and rare use of pedicled nasoseptal flaps (2.2% in this cohort), also likely contribute to the absence of permanent anosmia in this series [15, 26, 40]. While patients typically have transient hyposmia or anosmia during the first 1–4 weeks post-surgery, likely resulting from mucosal swelling and surgical manipulation, olfaction almost always fully recovers and is likely hastened by daily sino-nasal care with physiological saline irrigations and at least 2–3 endonasal debridements performed in the first 6 weeks post-surgery [15, 40].

This current series is unique in two aspects. First, there are few cohort studies with over 500 patients treated with the endoscopic endonasal approach. Second, there are no series to our knowledge that have analyzed major complication rates, LOS, readmissions and reoperations relative to the use of these specific complication avoidance protocols of (1) rescue flap approach, (2) Doppler probe for ICA localization, (3) pseudocapsular dissection and gland sparring techniques, and (4) graded repair of CSF leaks (Fig. 1). Our data suggests this protocolized approach is associated with low major complication rates of < 1% (for death, stroke, visual worsening, other neurological deficits, sellar hematoma, meningitis and anosmia), CSF leaks rates of under 2% and new hypopituitarism of under 5% (both of which are similar or lower than prior cohort studies) [33–38, 41–43]. Infrequent complications in the present series were also associated with a median LOS of 2 days and a 30-day readmission rate of 6%, similar to recent cohort studies but considerably shorter than national database studies [44–48]. Other studies have shown complication rates correlate inversely with hospital and surgical team experience [12, 43, 49–52]. Similarly, reoperations and readmissions (which was 10.4% in a recent national database study) are linked to complications, particularly hyponatremia and postoperative CSF rhinorrhea, and as our experience confirms, are linked to longer LOS [44–48].

### The learning curve

Our study and others show that even with a relatively large experience in endoscopic pituitary surgery, the learning curve continues and there remains room for improvement with further opportunity to reduce complications and shorten LOS [35, 53, 54]. For example, we were able to reduce our postoperative CSF leak rate to 1.2% and had no cases of meningitis in the second half of this series. This trend of decreased CSF leaks and meningitis was also demonstrated in our 2017 report on graded repair of CSF leaks in endoscopic surgery [26].

### Further optimizing outcomes in pituitary surgery with protocol-driven care

With increasing scrutiny of quality metrics, LOS and cost of care, health care economics will increasingly dictate a preference for a *“centers of excellence”* model, that relies on multidisciplinary expertise serving a high volume of patients [12, 13, 55–57]. Achieving optimal outcomes with high resection and remission rates, rare complications and few readmissions appears possible when an experienced team adhere to protocols for (1) avoiding vascular and gland injury, (2) achieving maximal tumor removal without causing new deficits of gland function or neurological deficits, (3) using reliable skull base closure techniques to avoid post-operative CSF leaks, (4) avoiding new postoperative anosmia, epistaxis and preserving sinonasal mucociliary function (5) promoting rapid mobilization, and discharge of patients, and (6) providing consistent and easy to follow discharge instructions on fluid intake and physical activity that reduce chances for delayed hyponatremia and CSF leak/meningitis [28]. The recent report by Burke et al. highlight the potential benefit of a strict outpatient protocol of fluid restriction for virtually eliminating readmissions for hyponatremia. In the current study, we had 18 readmissions for hyponatremia over 10 years; however, since launching a similar protocol in 2018, we have had only one such readmission in May 2018. The combination of a consistent post-discharge regimen for fluid restriction, liberal salt use and salted drinks in the first week post-surgery, as well as assessing serum sodium on post-operative day 5–7 appears to be effective in preventing and/or blunting most cases of delayed hyponatremia before a patient requires hospital readmission [58]. However, given that some patients leave the hospital in DI (12.1% transient and 2.3% permanent in this cohort) and are prescribed DDAVP as needed, individualized care and close team contact is essential for managing fluid balance especially in the first 2 weeks post-surgery.

Regarding length of stay, over the last year, in-part, driven by the COVID-19 pandemic-induced scarcity of monitored beds, we reduced our overall endonasal surgery median LOS to 1 day (including all tumor pathologies). We have also developed a protocol for discharge home the day of surgery in select patients. Same-day endoscopic pituitary surgery may be increasingly possible if complication avoidance protocols are adhered to strictly, and patients are carefully selected, informed and engaged [59].

Further improvements in outcomes, complications, length of stay and readmission may be possible with implementation of enhanced recovery after surgery (ERAS) protocols including reduced narcotic use [60–62]. A continued focus in neurosurgery, otolaryngology and endocrinology residency and fellowship training programs, emphasizing comprehensive patient management may also help achieve more consistent and broader national improvements in pituitary surgery outcomes [12, 45].

### Study limitations

This retrospective assessment is limited to the primary and secondary outcomes at 3 months post-surgery. As such, some patients with initial GTR or endocrine remission may have tumor progression over longer follow-up. Second, a minority of patients (5.6%) had missing data for hormonal recovery, mostly prior to converting to EPIC electronic medical record in 2014. Finally, while this analysis shows apparent utility of these complication avoidance protocols, these are only associations of outcomes in the setting of our treatment paradigm; there is no comparison cohort managed without these techniques and care model.

## Conclusion

Our experience suggests that protocol-driven endoscopic pituitary adenoma surgery performed at a high-volume center with a dedicated neurosurgical-otolaryngology team, can be associated with low rates of major complications and a short LOS, along with high rates of pituitary gland preservation and resection/remission rates. However, even with a large prior experience, the learning curve continues, and improvements can continually be made in the team management of pituitary adenomas to optimize outcomes and reduce complications.
